# Patch Testing in Individuals With Diabetes Using Medical Devices. Part 1—Contact Allergy to Baseline Series Allergens

**DOI:** 10.1111/cod.70117

**Published:** 2026-02-20

**Authors:** Thanisorn Sukakul, Josefin Ulriksdotter, Martin Mowitz, Magnus Bruze, Nils Hamnerius, Cecilia Svedman

**Affiliations:** ^1^ Department of Occupational and Environmental Dermatology Lund University, Skåne University Hospital Malmö Sweden

**Keywords:** contact allergy, continuous glucose monitoring, insulin infusion systems, type 1 diabetes

## Abstract

**Background:**

The prevalence of contact allergy in individuals with diabetes and diabetes medical device (MD) users is unknown.

**Objectives:**

This study (Part 1 and 2) aims at describing contact allergy prevalences in diabetes MD users.

**Methods:**

Adults with type 1 diabetes from two endocrinology departments were patch tested with the Swedish baseline series (SBS) and a MD series. Contact allergies to the baseline series were compared with consecutive dermatitis patients. Detailed results for the MD series are presented in another manuscript.

**Results:**

Overall, similar contact allergy prevalences to the SBS were seen in the 204 individuals with diabetes (114 with rash to diabetes MDs, 90 without) and 1382 controls (34.3% vs. 39.6%, adjusted *p* value = 0.30). The prevalence of sesquiterpene lactone mix allergy was higher in the individuals with diabetes vs. dermatitis controls (2.5% vs. 0.3%, adjusted *p* value = 0.0011). Contact allergy to *Myroxylon pereirae* resin (MP) was overrepresented in individuals with diabetes versus controls (8.3% vs. 3.8%, adjusted *p* value = 0.0033) but not in individuals with diabetes with rash to diabetes MDs vs. without.

**Conclusions:**

Contact allergy to the SBS is common in diabetes MD users. The cause of the overrepresentation of MP allergy needs to be further elucidated.

## Graphical abstract

The contact allergy prevalence in individuals with diabetes and in diabetes medical device (MD) users is unknown. This study in 204 diabetes MD users (114 with rash to diabetes MDs, 90 without) aimed at investigating the contact allergy prevalence to baseline series allergens. Contact allergies to sesquiterpene lactone mix and *myroxylon pereirae* resin (MP) were overrepresented in individuals with diabetes compared to consecutive dermatitis patients. Contact allergy to MP and overall fragrances was not related to rash to diabetes MDs and the cause of these allergies needs to be further investigated.

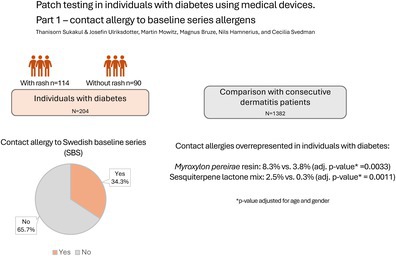

## 1 Introduction

Worldwide, contact dermatitis to diabetes medical devices (MDs) has during the past decade become a common problem in individuals with diabetes due to the increasing use of diabetes MDs including continuous glucose monitors (CGM) and devices for continuous insulin infusion (CSII). However, only a minority of the affected individuals is referred for patch testing [1]. The prevalence of contact allergy in general in individuals with diabetes type 1 is not known and neither is the prevalence of contact allergy to MD‐related allergens and allergic contact dermatitis (ACD) in the group using these [2]. The prevalence of MD‐related contact allergy might be underestimated due to limited access to patch testing and the limited availability of patch test substances [3]. The adverse skin reactions might be misdiagnosed as atopic dermatitis (AD), irritation or irritant contact dermatitis [4]. On the other hand, previously published data on prevalences of contact allergy to diabetes MDs in individuals referred for patch testing might also have overestimated the prevalence as the cases referred already are suspected to have a MD‐related contact allergy.

For dermatologists, dealing with skin reactions and performing contact allergy investigations or patch testing in this patient group are still challenging. There are no guidelines or recommendations on patch testing in individuals with suspected contact allergies to diabetes MDs. Isobornyl acrylate (IBOA) and colophonium are well‐known allergens among dermatologists as causes of ACD to diabetes MDs [5, 6, 7, 8, 9, 10, 11, 12] and patch test preparations of these substances are commercially available. However, due to product development and the variety of materials/chemicals used in MDs several additional allergens have been reported that are not always available for patch testing or the commercially available substances are not optimised and hence do not suffice [13, 14, 15, 16].

Although patients with adverse skin reactions to diabetes MDs are usually referred for aimed patch testing, they are also usually tested with the baseline series. Previous studies have reported that diabetes patients with suspected contact allergies to diabetes MDs might have other concomitant contact allergies [17, 18]. However, no evidence from a large or well‐designed study has been published.

Diabetes type 1 is a disease affecting both children and adults. The patients are increasingly being provided with MDs for prolonged use on the skin, a dermatological risk as such, but we do not have information on whether this group with the disease per se is prone to contact allergy due to exposures or possibly genetically. This diabetes MD contact allergy study was conducted to increase the understanding of contact allergies in individuals with diabetes using diabetes MDs and optimise dermatological investigations of those suspected of having MD contact allergy. This study comprises two parts as follows: (i) patch testing with baseline series/common screening allergens (presented in this manuscript, Part 1) and (ii) patch testing with a MD series (presented in a separate manuscript; Part 2 [19]). These two studies are the first cross‐sectional studies on contact allergy in individuals with type 1 diabetes who have been exposed to diabetes MDs, aiming at reporting the demographics and detailed results of patch testing, in a population not limited to referred cases with adverse skin reactions.

## 2 Materials and Methods

This cross‐sectional study (Part 1 and Part 2 [19]) was performed in 2021–2022 by the Department of Occupational and Environmental Dermatology, Skåne University Hospital, Malmö, Sweden (DOED). The investigations were conducted in southern Sweden in Halmstad (Region Halland) and Växjö (Region Kronoberg). The study was approved by the Swedish Ethical Review Authority, dnr 2020‐03160.

### 2.1 Participants

#### 2.1.1 Individuals With Diabetes

All adult individuals with type 1 diabetes (≥ 18 years) followed up at two outpatient diabetes clinics in The Southern Healthcare Region in Sweden (Halmstad and Växjö) were invited to participate in an online questionnaire concerning demographics, medical history, and adverse skin reactions associated with their diabetes MD use. The results of the questionnaire have been published elsewhere [1]. All respondents (*n* = 667) were invited to participate in the patch test study. Individuals with diabetes with ongoing immunomodulating medications, known pregnancy and those who had not used diabetes MDs were not included. The respondents who agreed to participate were invited to visit the clinics in Halmstad and Växjö for patch testing. Data on skin rash to diabetes MDs were retrieved from the questionnaire. Individuals with diabetes who were patch tested were subcategorized into two groups: (1a) participants who had experienced skin rash and (1b) those who had not experienced skin rash under the diabetes MDs. Written consent for participation was obtained from all participants.

#### 2.1.2 Dermatitis Patients

Adult (≥ 18 years) dermatitis patients routinely patch tested with the Swedish baseline and extended baseline series at DOED in Malmö during the same period were included as controls. Dermatitis patients who were tested with the MD series due to suspected contact allergy to MDs were excluded. Oral consents regarding the use of their anonymized patch test data for research and publication purposes were obtained from the dermatitis patients and documented in the patch test database.

### 2.2 Patch Testing

All participants were patch tested with the Swedish baseline series [20] which includes 29 test preparations and a MD patch test series. The allergens in the Swedish baseline series were categorised into allergen groups. Nickel (II) sulfate hexahydrate, cobalt (II) chloride hexahydrate and potassium dichromate were classified as metal allergens. Colophonium, fragrance mix I, II, lichen acid mix and *Myroxylon pereirae* resin (MP) were grouped as fragrance allergens. Methylchloroisothiazolinone/methylisothiazolinone, formaldehyde, paraben mix, diazolidinyl urea, methyldibromoglutaronitrile and quaternium 15 were classified as preservatives. Rubber allergens included mercapto mix, black rubber mix and thiuram mix. The prevalence of contact allergy to an allergen group was reported as a proportion of participants that reacted positively to at least one of the allergens in a group.

**FIGURE 1:**
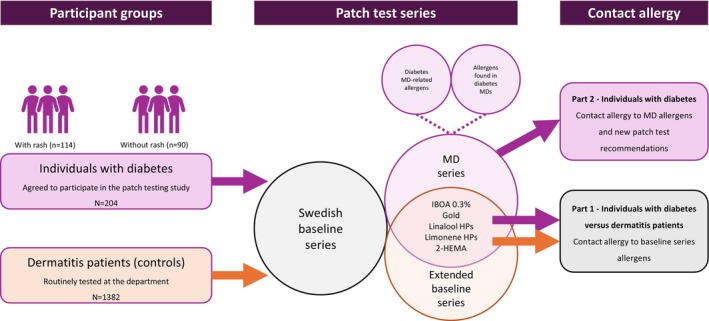
Participant groups and patch test series. Abbreviations: 2‐HEMA, 2‐hydroxyethyl methacrylate; HPs, hydroperoxides; IBOA, isobornyl acrylate; MD, medical device; N, number.

For the individuals with diabetes, a novel MD patch test series was additionally tested. The details of allergens included in the MD series are described in Part 2 [19]. Some allergens in the MD series are also patch tested in the department's extended baseline series, used for routine patch testing in consecutive dermatitis patients (Figure 1). They were included in the MD series due to specific reasons as follows. Additional fragrance allergens (not included in the Swedish baseline series) were included as fragrance contact allergy has been reported to be overrepresented in diabetes patients with adverse skin reactions to diabetes MDs [17, 18]. Gold may be used in CGM electrodes [21]. 2‐Hydroxyethyl methacrylate is considered as a screening allergen for (meth)acrylate allergy [22]. IBOA, the main allergen causing contact allergy to diabetes MDs, was also tested at the concentration 0.3% w/w in petrolatum in both individuals with diabetes and control dermatitis patients, whereas IBOA at 0.1% in petrolatum was tested only in individuals with diabetes.

The individuals with diabetes were patch tested with IQ Ultimate chambers (Chemotechnique Diagnostics, Vellinge, Sweden), whereas the controls were tested with Finn Chambers Aqua (SmartPractice, Phoenix, AZ, USA). The chambers were occluded on the back on Day 0 for 48 h. For IQ Ultimate chambers, 25 mg (39 mg/cm^2^) of the petrolatum preparations and 20 μL (31 μL/cm^2^) of liquid preparations were applied, whereas 20 mg (40 mg/cm^2^) of the petrolatum preparations and 15 μL (30 μL/cm^2^) of the liquid preparations were applied on the Finn Chambers Aqua [23, 24]. All commercially available patch test preparations were purchased from Chemotechnique Diagnostics, Vellinge, Sweden. Other patch test chemicals in the MD series were purchased and prepared in‐house at our laboratory (see details in Part 2 [19]). Patch test readings were performed on Day 3 or 4 and Day 7. The tests were read and scored according to the International Contact Dermatitis Research Group criteria [23, 25].

### 2.3 Statistical Analysis

Demographic data and prevalences of contact allergy were compared between groups; (1) the diabetes group further subclassified into (1a) a group with rash under CGM/CSII/infusion set (diabetes MDs) and (1b) a group without rash under diabetes MDs and (2) the dermatitis patients.

SPSS (version 29.0, IBM, New York, USA) was used for statistical analysis. Categorical data such as gender, a history of AD and patch test results are presented as absolute numbers and percentages. Age as continuous data is presented as mean and standard deviation. To report the statistical differences, categorical data was analysed using a two‐sided Chi‐square test or Fisher's Exact test, and continuous data (age) was analysed using a *t*‐test. For each analysis, data points with missing values were excluded from that particular analysis (such as unknown history of AD or incomplete patch test results for the individual patch test preparations). Multiple logistic regression analysis was used to compare the contact allergy prevalences between two groups. In the multivariable logistic regression analysis, adjustments were made for age group (18–49 and ≥ 50 years) and gender, since there were significant differences in age and gender between the individuals with diabetes and the dermatitis patients. Having a history of AD was not included in the multivariable regression analysis due to missing data in part of the dermatitis patients. For all analyses, *p* values less than 0.05 were considered statistically significant.

## 3 Results

### 3.1 Demographic Data

In this study, 1586 (66.7% females) were patch tested during the study period, 204 were individuals with diabetes (103 tested in Halmstad and 101 tested in Växjö), and 1382 were dermatitis patients tested in Malmö. Among the 204 individuals with type 1 diabetes, 114 (55.9%) reported to have had skin rash under diabetes MDs at some point. A comparison of the demographics between the groups is shown in Table 1. The mean age was significantly higher in individuals with diabetes than in the dermatitis patients (50.1 ± 17.6 vs. 44.0 ± 16.0 years; *p* < 0.001). In the individuals with diabetes (Group 1a/b), the proportion of males was higher than in the dermatitis patients (Group 2) and the self‐reported prevalence of a childhood AD was significantly lower (*p* value = 0.0023) than the prevalence of AD in the control dermatitis group (Group 2).

**TABLE 1:** Demographic data of the participants.

	Total	Individuals with diabetes			Dermatitis patients	*p* ^a^			
	*N* = 1586	Total *N* = 204	With rash *n* = 114	Without rash *n* = 90	*N* = 1382	DM versus dermatitis	DM rash versus DM no rash	DM rash versus dermatitis	DM no rash versus dermatitis
	*N* (%)	*N* (%)	*N* (%)	*N* (%)	*N* (%)				
Age (mean ± SD)	44.8 +/− 16.3	50.1 +/− 17.6	44.2 +/− 15.6	57.6 +/− 17.3	44.0 +/− 16.0	< 0.001	< 0.001	0.88	< 0.001
Age group						< 0.001	< 0.001	0.93	< 0.001
18–49 years	951 (60.0)	96 (47.1)	71 (62.3)	25 (27.8)	855 (61.9)				
≥ 50 years	635 (40.0)	108 (52.9)	43 (37.7)	65 (72.2)	527 (38.1)				
Gender						< 0.001	0.25	0.011	< 0.001
Female	1058 (66.7)	109 (53.4)	65 (57.0)	44 (48.9)	949 (68.7)				
Male	528 (33.3)	95 (46.6)	49 (43.0)	46 (51.1)	433 (31.3)				
Atopic dermatitis^b^ (*n* = 1376)	465 (33.8)	44/184 (23.9)	30/106 (28.3)	14/78 (17.9)	421/1192 (35.3)	0.0023	0.10	0.15	0.0017

Abbreviations: DM no rash, individuals with diabetes without rash to diabetes medical devices; DM rash, individuals with diabetes with rash to diabetes medical devices; DM, individuals with diabetes; *N*, number; SD, standard deviation.

^a^ Chi‐square test was used.

^b^ For the individuals with diabetes, the self‐reported prevalence of childhood atopic dermatitis and for the dermatitis patients, the physician‐diagnosed lifetime prevalence of atopic dermatitis.

### 3.2 Prevalence of Contact Allergy

In Table 2, the overall prevalences of contact allergy to the Swedish baseline series and allergen groups are presented. For individuals with diabetes, testing with the Swedish baseline series detected contact allergy in 34.3%. When testing with both the Swedish baseline series and the MD series, the prevalence of contact allergy among the individuals with diabetes was 52.5%.

**TABLE 2:** Comparison of the prevalence of contact allergy to the Swedish baseline series and allergen groups between the individuals with diabetes and the dermatitis patients.

	Individuals with diabetes			Dermatitis patients		Adjusted *p* ^a^			
	Total *N* = 204	With rash *N* = 114	Without rash *N* = 90	*N* tested	Positive	DM versus dermatitis	DM rash versus DM no rash	DM rash versus dermatitis	DM no rash versus dermatitis
	*N* (%)	*N* (%)	*N* (%)		*N* (%)				
Patch test results									
CA to at least 1 allergen in Swedish baseline series	70 (34.3)	44 (38.6)	26 (28.9)	1358	538 (39.6)	0.30	0.51	0.86	0.14
Fragrance contact allergy^b^	29 (14.2)	16 (14.0)	13 (14.4)	1372	165 (12.0)	0.29	0.61	0.56	0.32
Metal contact allergy^c^	32 (15.7)	21 (18.4)	11 (12.2)	1363	273 (20.0)	0.27	0.84	0.79	0.13
Rubber contact allergy^d^	1 (0.5)	0	1 (1.1)	1376	23 (1.7)	0.19	> 0.999	0.998	0.70
Preservative contact allergy^e^	8 (3.9)	4 (3.5)	4 (4.4)	1371	121 (8.8)	0.022*	0.47	0.062	0.15

Abbreviations: CA, contact allergy; DM no rash, individuals with diabetes without rash to diabetes medical devices; DM rash, individuals with diabetes with rash to diabetes medical devices; DM, individuals with diabetes; *N*, number.

^a^ Multiple logistic regression analysis adjusted for age (age group) and gender.

^b^ Contact allergy to fragrance mix I, fragrance mix II, *Myroxylon pereirae* resin, colophonium and/or lichen acid mix.

^c^ Contact allergy to nickel (II) sulfate hexahydrate, cobalt (II) chloride hexahydrate and/or potassium dichromate.

^d^ Contact allergy to mercapto mix, black rubber mix and/or thiuram mix.

^e^ Contact allergy to methylchloroisothiazolinone/methylisothiazolinone, formaldehyde, paraben mix, diazolidinyl urea, methyldibromoglutaronitrile and/or quaternium 15.

* Odds ratio (OR) (95% confidence interval (CI)) = 2.37 (1.13–4.94).

#### 3.2.1 Comparisons With the Control Dermatitis Patients

There was no significant difference in the overall prevalence of contact allergy to the Swedish baseline series between individuals with diabetes and dermatitis patients (Group 1a/b vs. 2) (34.3% vs. 39.6%, adjusted *p* value = 0.30).

### 3.3 Allergen Groups

Contact allergies to the different allergen groups are presented in Table 2. The prevalence of preservative allergy was significantly lower in the individuals with diabetes than in the dermatitis patients (Group 1a/b vs. 2, adjusted *p* value = 0.022). For the other allergen groups, no significant differences in the prevalence of contact allergy were seen when comparing individuals with diabetes with the dermatitis patients (Group 1a/b vs. 2).

### 3.4 Individual Allergens in Baseline Series

**FIGURE 2:**
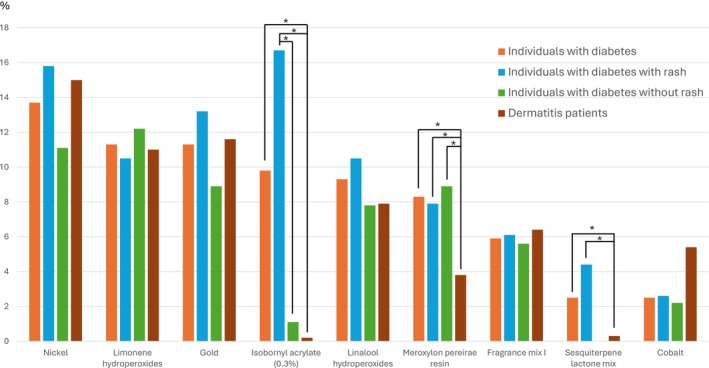
Most prevalent contact allergies^a^ among the individuals with diabetes (rash vs. no rash), compared with dermatitis patients. ^a^Contact allergy prevalence > 2% in the individuals with diabetes. *Adjusted *p* value < 0.05.

Table 3 and Figure 2 demonstrate the prevalence of contact allergy to the individual patch test preparations in the Swedish baseline series and selected allergens from the department's extended baseline series. The most common contact allergies seen to the allergens in the Swedish baseline series among individuals with diabetes (Group 1a/b) were nickel (II) sulfate hexahydrate (13.7%), MP (8.3%), fragrance mix I (5.9%), sesquiterpene lactone mix (SLM) (2.5%) and cobalt (II) chloride hexahydrate (2.5%). The prevalences of contact allergy to MP and SLM were significantly higher in the individuals with diabetes than in the dermatitis patients (Group 1a/b vs. 2, adjusted *p* values = 0.0033 and 0.0011, respectively (Table 3)).

**TABLE 3:** Comparison of the prevalence of contact allergy to the *individual test preparations* in the Swedish baseline series and additional relevant allergens between the individuals with diabetes and the dermatitis patients.

	Individuals with diabetes			Dermatitis patients		Adjusted *p* ^a^			
	Total*N* = 204	With rash *N* = 114	Without rash *N* = 90	Patients tested	Positive	DM versus dermatitis	DM rash versus DM no rash	DM rash versus dermatitis	DM no rash versus dermatitis
	*N* (%)	*N* (%)	*N* (%)	*N*	*N* (%)				
Allergens in baseline series									
Nickel(II)sulfate hexahydrate 5.0% pet.	28 (13.7)	18 (15.8)	10 (11.1)	1362	204 (15.0)	0.84	0.91	0.746	0.39
*Myroxylon pereirae* resin 25% pet.	17 (8.3)	9 (7.9)	8 (8.9)	1373	52 (3.8)	0.0033*	0.96	0.036*	0.013*
Fragrance mix I 8.0% pet.	12 (5.9)	7 (6.1)	5 (5.6)	1374	88 (6.4)	0.87	0.69	0.878	0.922
Sesquiterpene lactone mix 0.1% pet.	5 (2.5)	5 (4.4)	0	1377	4 (0.3)	0.0011*	0.999	< 0.001*	0.999
Cobalt(II)chloride hexahydrate 1.0% pet.	5 (2.5)	3 (2.6)	2 (2.2)	1370	74 (5.4)	0.11	0.71	0.247	0.26
Formaldehyde 2.0% aq.	4 (2.0)	2 (1.8)	2 (2.2)	1375	44 (3.2)	0.37	0.77	0.425	0.65
MCI/MI 0.215% aq.	4 (2.0)	1 (0.9)	3 (3.3)	1371	59 (4.3)	0.099	0.095	0.107	0.52
Fragrance mix II 14% pet.	3 (1.5)	1 (0.9)	2 (2.2)	1376	36 (2.6)	0.36	0.30	0.287	0.86
Phenol formaldehyde resin 2 1.0% pet.	3 (1.5)	1 (0.9)	2 (2.2)	1378	18 (1.3)	0.81	0.50	0.766	0.48
Colophonium 20% pet.	2 (1.0)	2 (1.8)	0	1376	42 (3.1)	0.14	0.999	0.425	0.998
Epoxy resin, Bisphenol A 1.0% pet.	2 (1.0)	2 (1.8)	0	1375	12 (0.9)	0.94	> 0.999	0.341	0.999
Textile dye mix 6.6% pet.	2 (1.0)	1 (0.9)	1 (1.1)	1375	27 (2.0)	0.30	0.77	0.403	0.53
Caine mix II 10% pet.	2 (1.0)	0	2 (2.2)	1377	20 (1.5)	0.66	0.999	0.998	0.44
PTBFR 1.0% pet.	1 (0.5)	1 (0.9)	0	1378	14 (1.0)	0.58	> 0.999	0.876	0.999
Potassium dichromate 0.5% pet.	1 (0.5)	1 (0.9)	0	1373	54 (3.9)	0.039*	> 0.999	0.137	0.997
Diazolidinyl urea 2.0% aq.	1 (0.5)	1 (0.9)	0	1378	7 (0.5)	0.89	> 0.999	0.537	0.999
Lichen acid mix 0.3% pet.	1 (0.5)	0	1 (1.1)	1377	9 (0.7)	0.67	> 0.999	0.999	0.76
*p*‐phenylenediamine 1.0% pet.	1 (0.5)	0	1 (1.1)	1363	23 (1.7)	0.21	> 0.999	0.998	0.61
Amerchol L‐101 50% pet.	1 (0.5)	0	1 (1.1)	1377	6 (0.4)	0.82	> 0.999	0.999	0.20
Black rubber mix 0.6% pet.	1 (0.5)	0	1 (1.1)	1377	4 (0.3)	0.64	> 0.999	0.999	0.19
Budesonide 0.01% pet.	1 (0.5)	0	1 (1.1)	1373	17 (1.2)	0.41	> 0.999	0.998	0.93
Quinoline mix 6.0% pet.	0	0	0	1377	6 (0.4)	0.999	NA	0.999	0.999
Mercapto mix 3.5% pet.	0	0	0	1381	4 (0.3)	0.999	NA	0.999	0.999
Thiuram mix 1.0% pet.	0	0	0	1377	16 (1.2)	0.998	NA	0.998	0.999
Paraben mix 16% pet.	0	0	0	1377	5 (0.4)	0.999	NA	0.999	0.999
Neomycin sulfate 20% pet.	0	0	0	1374	7 (0.5)	0.999	NA	0.999	0.999
Tixocortol‐21‐pivalate 0.1% pet.	0	0	0	1376	14 (1.0)	0.999	NA	0.999	0.999
Quaternium‐15 1.0% pet.	0	0	0	1375	14 (1.0)	0.999	NA	0.999	0.999
MDBGN 0.5% pet.	0	0	0	1376	20 (1.5)	0.998	NA	0.998	0.998
Allergens in extended baseline series**									
Hydroperoxides of limonene 0.3% pet.	23 (11.3)	12 (10.5)	11 (12.2)	1332	146 (11.0)	0.38	0.71	0.96	0.16
Gold (I) sodium thiosulfate dihydrate 2.0% pet.	23 (11.3)	15 (13.2)	8 (8.9)	1336	155 (11.6)	0.67	0.58	0.34	0.60
Isobornyl acrylate 0.3% pet.	20 (9.8)	19 (16.7)	1 (1.1)	1331	3 (0.2)	< 0.001*	0.0045*	< 0.001*	0.31
Hydroperoxides of linalool 1.0% pet.	19 (9.3)	12 (10.5)	7 (7.8)	1308	103 (7.9)	0.33	0.36	0.31	0.67
2‐Hydroxyethyl methacrylate 2.0% pet.	0	0	0	1335	19 (1.4)	0.998	NA	0.998	0.998

Abbreviations: aq, aqua; DM, individuals with diabetes; DM no rash, individuals with diabetes without rash to diabetes medical devices; DM rash, individuals with diabetes with rash to diabetes medical devices; MCI/MI, methylchloroisothiazolinone/methylisothiazolinone; MDBGN, Methyldibromo glutaronitrile; NA, not applicable; N, number; pet., petrolatum; PTBFR, 4‐tert‐butylphenolformaldehyde resin.

^a^ Multiple logistic regression analysis adjusted for age (age group) and gender.

* Statistically significant (*p* value < 0.05).

** Tested in consecutive dermatitis patients but not included in the Swedish baseline series.

For allergens in the extended baseline series, a high prevalence of contact allergy to gold (I) sodium thiosulfate dihydrate (11.3%), hydroperoxides of limonene (11.3%) and hydroperoxides of linalool (9.3%) was seen in the individuals with diabetes (Group 1a/b). However, the contact allergy prevalences for these allergens were not significantly different from the dermatitis patients (Group 2). A positive reaction to IBOA 0.3% in petrolatum was seen in 9.8% of the individuals with diabetes and 0.2% of the controls (Group 1a/b vs. 2, adjusted *p* value < 0.001). None of the individuals with diabetes reacted to 2‐hydroxyethyl methacrylate 2.0% in petrolatum.

#### 3.4.1 Contact Allergies in Subgroups of Individuals With Diabetes

Individuals with diabetes with rash (Group 1a) had a numerically higher prevalence of contact allergy to the baseline series (38.6% vs. 28.9%, adjusted *p* value = 0.51) and metals (18.4% vs. 12.2%, adjusted *p* value = 0.84) than individuals with diabetes without rash to diabetes MDs (Group 1b). A similar prevalence of fragrance and preservative contact allergy was observed in individuals with diabetes with rash and without rash to diabetes MDs (Group 1a vs. 1b). A significantly higher prevalence of contact allergy to IBOA was seen in those with rash than without rash to diabetes MDs (Group 1a vs. 1b), adjusted (*p* value = 0.0045). Furthermore, all 5 SLM positive individuals with diabetes were positive to IBOA and had experienced rash to diabetes MDs.

When comparing the individuals with diabetes that were patch tested in Halmstad versus Växjö there was found no significant differences in the prevalence of contact allergy to the Swedish baseline series (*p* value = 0.22). As mentioned, the details of patch testing with the MD series are described in Part 2 [19].

## 4 Discussion

Many cases of ACD to diabetes MDs and relevant culprit allergens in the devices have been reported. As could be expected in this study, higher prevalences of contact allergy to IBOA and SLM were seen in individuals with diabetes with rash than in those without rash to diabetes MDs. However, previously published studies have also reported on a higher than expected prevalence of fragrance allergy in diabetes patients with contact dermatitis [18] and contact allergy to diabetes MDs [17]. This study could confirm a high prevalence of fragrance allergy in the individuals with diabetes and a significantly higher prevalence of contact allergy to MP in the individuals with diabetes than in the dermatitis patients. However, there was no significant overrepresentation of MP allergy or fragrance allergy in the individuals with diabetes with rash compared to those without rash to diabetes MDs indicating that these allergies were not clearly associated with the MD‐related rash. Individuals with diabetes are not routinely patch tested neither before nor after treatment with diabetes MDs is initiated and the general contact allergy pattern among individuals with diabetes is not known. In this study, the contact allergies found are discussed and compared with previous studies. However, when comparing contact allergy prevalences across studies, the different patch test methods and procedures declared in the studies should always be taken into account.

Contact allergy to SLM has been reported to be overrepresented in individuals with diabetes with IBOA contact allergy [18, 26, 27]. This study has confirmed this finding, as all five individuals with diabetes that were positive to SLM had experienced rashes from their diabetes MDs and had simultaneous positive reactions to IBOA. On the other hand, all four dermatitis patients with SLM contact allergy did not have IBOA contact allergy (5/5 versus 0/4; *p* value = 0.0079, Fisher's exact test, 2‐sided). Therefore, IBOA cross‐sensitization may not be expected if an individual has been sensitised to SLM. This strongly suggests that individuals with diabetes and IBOA contact allergy were sensitised through exposure to MD adhesives containing IBOA, rather than being pre‐sensitised to SLM.

IBOA must be patch tested in individuals with skin rash to diabetes MDs but testing with IBOA cannot be recommended in individuals with diabetes who do not have rash to diabetes MDs or in general dermatitis patients [18, 28]. No irritant reactions and late‐appearing reactions appearing beyond D10 indicating active sensitization were reported in our patients tested with IBOA at the concentration of 0.3% (0.12 mg/cm^2^), which therefore can be considered safe for patch testing.

The overall prevalence of contact allergy to the Swedish baseline series in individuals with diabetes without rash to diabetes MDs (28.9%, Table 2) was in line with what has previously been reported for the general European population (27.0%) [29], which could be expected. The highest contact allergy prevalences in the individuals with diabetes were seen to metals and fragrances. However, the prevalences of contact allergy to nickel (13.7%) and cobalt (2.5%) among individuals with diabetes were similar to that in the general European population (14.5% and 2.2% [29], respectively). MDs are known to have metal components and suspected ACD to metal in a diabetes MD have been reported [30]. However, metals are not known to be the main culprit allergens in diabetes MDs.

Contact allergy to fragrance allergens in the Swedish baseline series in the individuals with diabetes (14.2%) was more common than in the general population (estimated prevalence 4%–5%) [29, 31]. Furthermore, the prevalence of MP allergy was significantly higher in the individuals with diabetes than in the dermatitis patients (adjusted *p* value = 0.0033).

Our findings are difficult to explain and will require further research. Individuals with diabetes using diabetes MDs might have skin barrier defects on the application site, which could possibly facilitate sensitization to fragrance allergens in topical skin products. However, in that case a similar pattern would have been expected for preservatives, and the low prevalence of preservative contact allergy could not support the hypothesis. Another possibility is that the fragrance allergies are caused by exposure to the diabetes MDs. MP resin ingredients and non‐oxidised limonene have been found in the adhesive parts of the MDs and thus might contribute to fragrance contact allergy [32, 33]. Furthermore, medical adhesives can be sources of exposure to colophonium, a fragrance contact allergy marker [34]. In this study, there were only two individuals with diabetes who had contact allergy to colophonium; hence this cannot explain the high rate of fragrance contact allergy. The data in this study were collected during a period when acrylates (especially IBOA), rather than colophonium, were the predominant culprit allergens identified in diabetes MDs. Therefore, a high prevalence of colophonium contact allergy due to sensitization from exposure in diabetes MDs could not be expected.

It should be noted that testing with 2‐hydroxyethyl methacrylate 2.0%, which is recommended to be tested in patients with suspected (meth)acrylate allergy [22], cannot be used as a screening allergen for acrylate allergy in individuals with diabetes with skin rash to diabetes MDs since none of the individuals with diabetes reacted to it and this allergen has not been found in the diabetes MDs [19].

### 4.1 Study Limitations

Using different patch test chambers in the individuals with diabetes and the dermatitis patients could be a limitation in this study as the prevalence of contact allergy to some allergens might differ when testing with different chamber systems [35, 36]. However, this should not affect the overall prevalence of contact allergy or prevalence of contact allergy to the allergen groups. According to the information retrieved from the questionnaire [1] some data might be missing, for example in patients with an unknown history of childhood AD, which was not included in the multivariable logistic regression analysis. For the individuals with diabetes, the self‐reported prevalence of childhood AD was reported and for the control dermatitis patients the lifetime prevalence of AD. This might in part explain the different prevalences of AD in the two groups.

## 5 Conclusions

Both a MD patch test series *and* a baseline series should be patch tested when suspecting contact allergy to diabetes MDs. Patch testing with IBOA is necessary in individuals with diabetes with rash to diabetes MDs but not in those without rash or in general dermatitis patients. MP contact allergy and overall fragrance allergy were not significantly associated with skin rash to diabetes MDs. The causes of the overrepresentation of MP allergy and overall high prevalence of fragrance allergy, sources of exposure and clinical relevance in this group should be further studied.

## Author Contributions

**Martin Mowitz:** conceptualization, investigation, methodology, writing – review and editing, data curation, supervision. **Magnus Bruze:** conceptualization, investigation, methodology, writing – review and editing, data curation, supervision. **Josefin Ulriksdotter:** conceptualization, investigation, writing – original draft, methodology, writing – review and editing, data curation. **Thanisorn Sukakul:** conceptualization, investigation, writing – original draft, methodology, writing – review and editing, formal analysis, data curation, supervision. **Cecilia Svedman:** conceptualization, investigation, methodology, writing – review and editing, data curation, supervision, resources. **Nils Hamnerius:** conceptualization, investigation, methodology, writing – review and editing, data curation, supervision.

## Funding

The Stig and Ragna Gorthon Foundation, Hudfonden, Svenska Diabetesstiftelsen, Swedish Asthma and Allergy Association's Research Foundation.

## Ethics Statement

The study was approved by the Swedish Ethical Review Authority, dnr 2020‐03160.

## Conflicts of Interest

M.B. is a member of a fragrance safety expert panel; http://fragrancesafetypanel.org/. C.S. participates in the Extended Fragrance Ingredients Surveillance Study (a fragrance study) that is performed on behalf of The International Fragrance Association. T.S. is a member of the IDEA working group on oxidised terpenes.

## Data Availability

The data underlying this article is available in the article and additional data can be provided upon reasonable request within the limits of the ethical approval.
